# Effect of Unplanned Therapy on the Prognosis of Patients with Extremity Osteosarcoma

**DOI:** 10.1038/srep38783

**Published:** 2016-12-08

**Authors:** Bing Wang, Ming Xu, Kai Zheng, Xiuchun Yu

**Affiliations:** 1 Orthopedic Department, The General Hospital of Jinan Military Commanding Region, Jinan, P.R.China

## Abstract

Unplanned therapy for extremity osteosarcoma can result in erroneous surgical procedures and lack of neoadjuvant chemotherapy before the first operation. Our aim was to compare the prognosis between patients with extremity osteosarcoma who received unplanned therapy and those who received standard treatment. This was a retrospective review of patients with extremity osteosarcoma who received appropriate surgical treatment and neoadjuvant chemotherapy (n = 79) and those who received unplanned therapy (n = 24) between June 2000 and October 2014. Survival rate, local recurrence rate and metastasis rate were compared between the two groups. We found that patients who had unplanned therapy had a higher local recurrence rate (41.7% vs. 21.5%; *P* = 0.049) and a shorter mean time for recurrence (8.90 vs. 14.59 months; *P* = 0.018). There was no significant difference between groups in the 5-year survival rate (56.3% vs.67.8%; *P* = 0.356), metastasis rate (45.8% vs. 30.4%; *P* = 0.125) and mean time to metastasis (23.18 vs.18.24 months; *P* = 0.396). Our findings suggest that unplanned therapy for extremity osteosarcoma can result in failure of local control. The use of supplementary interventions after unplanned therapy, such as neoadjuvant chemotherapy and limb salvage surgery, may explain the similar survival and metastasis rates between patients receiving unplanned therapy and those receiving standard treatment.

Osteosarcoma (OS) is one of the most prevalent malignant bone tumors affecting adolescents aged between 10 and 24 years, and the incidence of this disease is about 4/1000000^1^,^2^,^3^. Accurate diagnosis of high-grade OS requires the integration of clinical, radiological and histological data. The relatively low incidence of OS and limited experience in non-specialized centers likely accounts for instances of misdiagnosis and inappropriate treatment, including erroneous surgical procedures and a failure to administer neoadjuvant chemotherapy^4^,^5^,^6^. Such unplanned therapy, including operations with inadequate surgical margins and failure to use neoadjuvant chemotherapy before the first surgery, has been reported to negatively affect patient survival rate and local recurrence rate^7^. The main purpose of this retrospective study was to compare prognosis between patients who underwent unplanned management followed by salvage therapy and those who received standard treatment. A secondary aim was to review which salvage therapies had been administered to patients who received unplanned therapy.

## Materials and Methods

This was a retrospective and comparative study carried out at a single institution. The investigations were performed after approval by the Ethics Committee of the General Hospital of Jinan Military Commanding Region and the study was performed in accordance with relevant guidelines and regulations. All patients provided written informed consent. The study included 103 patients with extremity OS who were treated surgically between June 2000 and October 2014. The patients were divided into two groups. Group A included patients who were misdiagnosed in non-specialized centers and received inappropriate initial surgery without neoadjuvant chemotherapy (n = 24). Group B included patients for whom a pathological diagnosis was obtained by needle biopsy and who received appropriate surgical treatment and neoadjuvant chemotherapy (n = 79).

For the patients in Group A (who received unplanned therapy), the lesions were in the proximal tibia (n = 8), distal femur (n = 10), proximal femur (n = 3), distal tibia (n = 1), proximal fibula (n = 1) and proximal humerus (n = 1). The initial misdiagnosis and inappropriate surgery for the patients in Group A were as follows: osteomyelitis (n = 5; treated by flushing drainage in 2 cases and clearance of the focal lesion in 3 cases), bone cyst (n = 1; treatedby curettage with bone cement filling), fibrous dysplasia (n = 2; treatedby curettage with bone graft), soft tissue tumor (n = 3; treated by resection), common bone fracture (n = 4; treatedbyopen reduction with internal fixation), and unclear diagnosis (n = 9; treatedbyincision and exploration). None of the patients in Group A received neoadjuvant chemotherapy before the initial surgical treatment. The delay in the diagnosis and treatment of patients in Group A ranged from 5 days to 6 months (1.10 ± 1.55 months). The lesions in patients in Group B were in the proximal tibia (n = 25), distal femur (n = 41), proximal femur (n = 1), distal tibia (n = 1), proximal fibula (n = 6), proximal humerus (n = 4) and distal radius (n = 1; Table 1).

**Figure 1:**
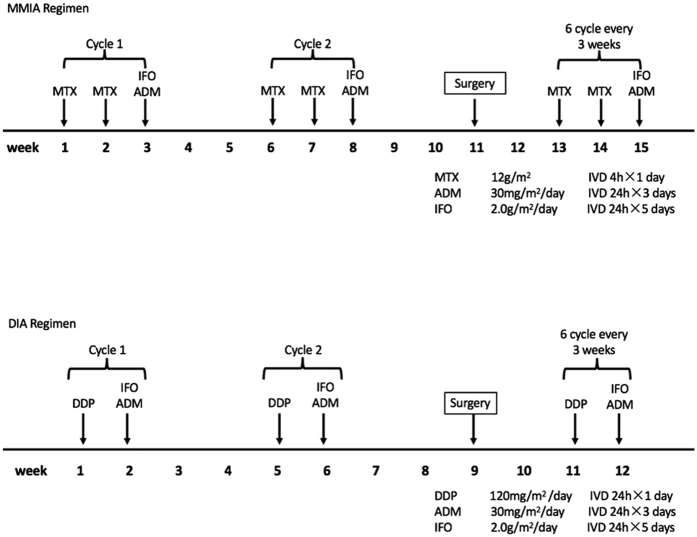
Neoadjuvant chemotherapy regimens. MTX, methotrexate; DDP, cisplatin; IFO, ifosfamide; ADM, adriamycin.

The patients in Group A were given neoadjuvant chemotherapy (using the same protocol as that for adjuvant therapy; Fig. 1) in our center and underwent surgical treatment, including a limb salvage procedure in 22 cases and amputation in 2 cases. The salvage surgery for patients in Group A included alcohol inactivated grafting (n = 9), *in situ* microwave inactivation (n = 2), wide resection (n = 1), wide resection of the tumor with fibula transplantation (n = 1), and prosthesis replacement (n = 9; of the knee in6 cases, of the hip in3 cases). All patients in Group B were administered neoadjuvant chemotherapy after OS had been confirmed by biopsy. Surgery for patients in Group B included a limb salvage procedure in 75 cases and amputation in 4 cases. The salvage surgery for patients in Group B included alcohol inactivated grafting (n = 37), *in situ* microwave inactivation (n = 7), wide resection (n = 6), wide resection with bone transport (n = 1), prosthesis replacement (n = 23; of the knee in22 cases, of the hip in 1 case), and massive allograft transplantation (n = 1).

All patients were treated with adjuvant chemotherapy after surgery, according to the protocol used at our institution at that time and consistent with protocols reported previously^8^,^9^. Two different regimens were used. In the MMIA regimen (used from June 2000 to July 2003; Group A, n = 7; Group B, n = 24), patients received a combination of 12 g/m^2^/day (high-dose) methotrexate, 2.0 g/m^2^/day ifosfamide and 30 mg/m^2^/day adriamycin. In the DIA regimen (used from July 2003 to October 2014; Group A, n = 17; Group B, n = 55), patients received a combination of 120 mg/m^2^/day cisplatin, 2.0 g/m^2^/day ifosfamide, and 30 mg/m^2^/day Adriamycin (Fig. 1).

For the identification of metastases and local recurrence, chest computed tomography scans and plain radiographs (and also magnetic resonance imaging if necessary) were obtained at the time of diagnosis, every month for the first half year postoperatively, every 3 months for the next 2 years, every 6 months during the third to fifth years, and then annually. Any observation of metastasis or local recurrence was recorded.

Fisher’s exact chi-squared test and Student’s t-test were used to compare the clinical characteristics between the two groups. The Kaplan-Meier method was used to compare survival between groups. All analyses were performed with SPSS software version 19.0 (SPSS Inc. IBM Co, USA). A statistically significant difference was defined as *P* < 0.05.

## Results

The average follow-up of the 103 patients was 60.46 months (range: 6–177 months). The symptom length ranged from 10 days to 3 months in Group A (unplanned therapy) and from 7 days to 5 months in Group B (standard treatment). There were no significant differences between the two groups in the male: female distribution (Table 1; *P* = 0.792), mean age (Table 1; *P* = 0.440), symptom length (*P* = 0.379) or pathological subtype (Table 1; *P* = 0.765); osteoblastic OS was the most common subtype in both groups, followed by chondroblastic OS (Table 1). The postoperative complications inpatients in Group A included2 cases of prosthesis loosening requiring revision surgery, 2 cases of incisional infections (1 case underwent amputation because of infection 4 years after surgery; 1 case was treated successfully with debridement and anti-infection therapy), and 1 case with inactivated bone fracture. The postoperative complications in patients in Group B included 3 cases of incisional infection requiring debridement and anti-infection therapy, 1 case of prosthesis requiring revision surgery, 1 case of internal fixation breakage treated with bone grafting and internal fixation surgery, 1 case of foot drop caused by common peroneal nerve injury, and 4 cases within activated bone fracture that underwent bone grafting.

**Figure 2:**
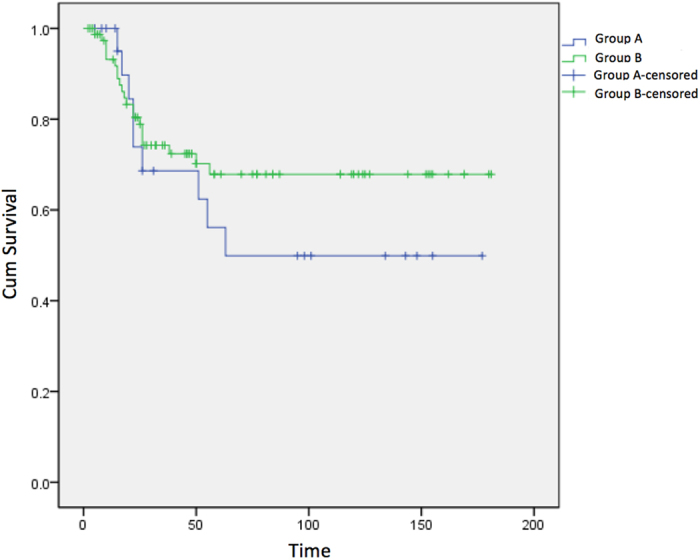
Kaplan-Meier survival curves for the two groups.

Ten patients (41.7%) in Group A (unplanned treatment) experienced local recurrence as compared with 17 patients (21.5%) in Group B (*P* = 0.049), and the mean time to local recurrence was shorter for Group A than for Group B (8.90 versus14.59 months; *P* = 0.018; Table 2). Lung metastasis occurred in 11 patients (45.8%) who received unplanned treatment and 24 patients (30.4%)who underwent planned therapy (*P* = 0.162); there was also no significant difference between groups in the mean time to lung metastasis (23.18 versus18.24 months; *P* = 0.396; Table 2). The 5-year survival rates were similar for patients who received unplanned treatment (56.3%) and those who received standard treatment (67.8%; *P* = 0.356; Table 2; Fig. 2).

## Discussion

Osteosarcoma was once considered a fatal disease, but following the successful development of chemotherapy in the early 1970’s there have been spectacular improvements in the 5-year survival rate, which can reach60–80%^10^,^11^,^12^,^13^. The standard treatment protocol for this disease includes the use of neoadjuvant chemotherapy and extensive surgical resection of the tumor^14^. The prognosis of patients with OS is affected by both tumor burden-related factors and treatment-related factors. The former include tumor volume and the presence of metastases, whereas the latter include the response to chemotherapy and the surgical margin^15^,^16^. Wang *et al*. found that a lack of neoadjuvant chemotherapy and inadequate surgical margins can result in failure of local control and earlier systemic tumor metastases in patients who received unplanned treatment for OS^4^. Our study observed that the local recurrence rate was significantly higher in patients who underwent unplanned primary surgery without neoadjuvant chemotherapy (37.5%) than in those who underwent standard treatment (21.9%). Furthermore, the mean time to local recurrence was shorter in the unplanned treatment group.

Factors known to influence local recurrence include the surgical margin, the site of the tumor and the response to chemotherapy^17^. Control of the surgical margin is particularly important. Unplanned surgery may result in inadequate surgical margins, inappropriate skin incision and inappropriate drainage locations. These factors may lead to a higher local recurrence rate and earlier local recurrence. However, due to the small sample size, it was not possible to assess the association between individual surgical methods and local recurrence rate in our study. Regardless of the surgical method used, destruction of the surgical margin likely led to local recurrence. The time from surgery to local recurrence may have been shorter in the unplanned treatment group because of the extensive surgery that these patients received and substantial contamination by the tumor during the initial unplanned surgery. Thus, incisional biopsy for histological diagnosis should be performed carefully, and core needle biopsy should be the preferred method^18^,^19^.

The lung is the most common site of OS metastasis. It has been reported that nearly 20% of patients have lung metastasis or small pulmonary nodulesat the time of the initial diagnosis^20^,^21^. Chemotherapy can effectively control the metastasis of OS^22^ and enhance the survival rate of patients with OS who have metastases^23^. In recent years, the importance of neoadjuvant chemotherapy for the control of metastasis has been confirmed^24^. The use of neoadjuvant chemotherapy without an ensuing treatment delay could improve the survival rate and limb salvage rate in patients who underwent unplanned treatment^25^,^26^. In the present study, the metastasis rate for patients who underwent unplanned surgery was not significantly higher than that for patients who received standard therapy (45.3% and 30.4%), and the 5-year survival rates were also not significantly different between the two groups (56.3% and 67.8%, respectively). One possible reason for this result is that the patients who underwent unplanned treatment subsequently received aggressive salvage therapy, including neoadjuvant chemotherapy and extensive surgical treatment. The use of this supplementary treatment may have prevented the unplanned treatment from having a detrimental effect on metastasis rate and 5-year survival. This would be consistent with a study by Jeon and colleagues, which found that limb salvage procedures in patients with osteosarcoma, initially treated with unplanned intralesional procedures, resulted in a disease-free 5-year survival comparable to that of patients receiving standard treatment^27^. However, another possible explanation for these observations is that our study was underpowered to detect a significant difference between the two groups; it was notable that, despite a lack of statistical significance, the metastasis rate was numerically higher and the 5-year survival rate numerically lower in the unplanned therapy group than in the standard treatment group. Thus, additional studies with larger sample sizes are needed to establish whether unplanned therapy in patients with OS is associated with a higher incidence of metastasis or a lower 5-year survival rate.

Since inappropriate and invasive surgery can result in a higher OS recurrence rate, patients with unplanned treatment are generally considered more likely to require amputation^28^. Although all 24 patients who received unplanned treatment underwent limb salvage surgery, two patients required amputation due to tumor invasion of blood vessels and nerves. Limb salvage should still be attempted whenever possible after the control of surgical margins and the application of neoadjuvant chemotherapy^7^,^26^.

Therefore, we advise that patients who have received unplanned initial surgery should be given standard neoadjuvant chemotherapy without delay as a supplementary therapy, and extensive surgical treatment, especially limb salvage surgery, should be considered in order to obtain appropriate surgical margins.

The present study has certain limitations hence its findings should be interpreted with a degree of caution. First, this was a retrospective study, thus it cannot be excluded that selection bias or reporting bias influenced our findings. Second, the number of cases included in this study was small, so the study may have been underpowered to detect real differences between groups for some variables. However, it should be noted that the sample size in our study compared favorably with previously published studies (Table 3). Third, this was a single-center study, so the results may not be generalizable to the general patient population in China or elsewhere. Fourth, patient age could potentially have influenced survival outcome, but the patients in our study were grouped together irrespective of age (although it should be noted that there was no significant difference in patient age between the two groups). The sample size in our study was too small to allow subgroup analysis based on patient age, but performing such an analysis will be important for future investigations. Prospective, large-scale, multi-center clinical studies are needed to confirm and extend our findings.

## Conclusions

Accurate initial diagnosis with the option of appropriate and timely surgery is important in the management OS of the extremities. Failure to administer neoadjuvant chemotherapy and inadequate surgical margins can lead to failure of local control in patients who have undergone unplanned treatment, increasing the local recurrence rate and reducing the time to local recurrence. However, it is possible that long-term survival rate and metastasis rate are not detrimentally affected in patients who receive unplanned treatment if supplementary therapies, i.e. standard neoadjuvant chemotherapy and limb salvage surgery, are subsequently administered in a sufficiently timely manner.

## Additional Information

**How to cite this article**: Wang, B. *et al*. Effect of Unplanned Therapy on the Prognosis of Patients with Extremity Osteosarcoma. *Sci. Rep.* **6**, 38783; doi: 10.1038/srep38783 (2016).

**Publisher's note:** Springer Nature remains neutral with regard to jurisdictional claims in published maps and institutional affiliations.

## Figures and Tables

**Table 1:** Baseline characteristics of the patients included in the analysis.

	Group A	Group B	*P* value
Gender			0.792
Male	15 (62.5%)	47 (59.5%)	
Female	9 (37.5%)	32 (40.5%)	
Age			0.440
≤15	9 (37.5%)	39 (49.4%)	
>15 and ≤40	14 (58.3%)	39 (49.4%)	
>40	1 (4.2%)	1 (1.3%)	
Osteosarcoma site			—
Distal tibia	1 (4.2%)	1 (1.3%)	
Proximal tibia	8 (33.3%)	25 (31.7%)	
Distal femur	10(41.7%)	41 (51.9%)	
Proximal femur	3 (12.5%)	1 (1.3%)	
Proximalfibula	1 (4.2%)	6 (7.6%)	
Proximal humerus	1 (4.2%)	4 (5.1%)	
Distal radius	0 (0)	1 (1.3%)	
Pathological subtype			
Osteoblastic	14 (58.3%)	39 (49.4%)	0.765
Chondroblastic	5 (20.8%)	16 (20.3%)	
Fibroblastic	2 (8.3%)	13 (16.5%)	
Other	3 (12.5%)	11 (13.9%)	
Chemotherapy			0.910
DIA	17 (70.8%)	55 (69.6%)	
MMIA	7 (29.2%)	24 (30.4%)	
Operation			0.549
Amputation	2 (8.3%)	4 (5.1%)	
Limb salvage	22 (91.7%)	75 (94.9%)	

**Table 2:** Comparison of outcome measures between the two groups.

	Group A	Group B	*P* value
Local recurrence			0.049
Yes	10 (41.7%)	17 (21.5%)	
No	14 (58.3%)	62 (78.5%)	
Time to recurrence (months)	8.90 ± 3.93	14.59 ± 7.68	0.018
Metastasis			0.162
Yes	11 (45.8%)	24 (30.4%)	
No	13 (54.2%)	55 (69.6%)	
Time tometastasis (months)	23.18 ± 16.96	18.24 ± 12.07	0.396
Survival rate			
5-year survival rate	56.3%	67.8%	0.356

**Table 3:** Literature review of articles researching unplanned treatment for OS.

Author	Cases	Local recurrence rate (%)	Metastasis rate (%)	Survivalrate (%)
Ayerza *et al*.^7^	9	55	NA	44.0
Kim *et al*.^23^	20	0	10	89.4
Wang *et al*.^4^	16	43.8	50	60.9
Jeon *et al*.^27^	22	18	24	65
Our study	24	41.7	45.8	56.3
